# Case report: Methylphenidate improved chronic pain in an adult patient with attention deficit hyperactivity disorder

**DOI:** 10.3389/fpsyt.2023.1091399

**Published:** 2023-03-10

**Authors:** Ekachaeryanti Zain, Atsunori Sugimoto, Jun Egawa, Toshiyuki Someya

**Affiliations:** ^1^Department of Psychiatry, Niigata University Graduate School of Medical and Dental Sciences, Niigata, Japan; ^2^Department of Psychiatry, Faculty of Medicine, Mulawarman University, Samarinda, Indonesia; ^3^Department of Community Psychiatric Medicine, Niigata University Graduate School of Medical and Dental Sciences, Niigata, Japan; ^4^Department of Psychiatry, Niigata Psychiatric Center, Nagaoka, Japan; ^5^Department of Psychiatry, Niigata University Medical and Dental Hospital, Niigata, Japan

**Keywords:** attention deficit hyperactivity disorder, adult ADHD, chronic pain, methylphenidate, case report

## Abstract

**Introduction:**

Chronic pain remains a health problem that is difficult to treat adequately. Its unknown cause and complex comorbidity with other illnesses, including mental disorders, amplify the severity of symptoms, which consequently decreases the quality of life of patients long term. In our clinical practice, we coincidentally found evidence that methylphenidate (MPH) effectively managed chronic pain in an adult patient with attention deficit hyperactivity disorder (ADHD). The effectiveness of MPH in the treatment of ADHD is well-established; however, its utility in treating pain remains unclear.

**Case presentation:**

We present a rare case of a 43-year-old male patient with 15 years of chronic idiopathic pain symptoms that did not adequately respond to standard pain management, such as acetaminophen, non-opioid analgesics, and muscle relaxers. Pain also persisted after treatments with antidepressants and an epidural block. Furthermore, symptoms worsened following several sessions of modified electroconvulsive therapy. After a thorough assessment at our child and adolescent psychiatric outpatient clinic, we confirmed a diagnosis of adult ADHD with a predominantly inattentive type. Considering this newly established diagnosis, we prescribed osmotic-release oral system (OROS) methylphenidate. Within 1 month of treatment at a dose of 18 mg/day of OROS-MPH, the patient’s chronic pain unexpectedly improved dramatically, and the patient no longer experienced pain symptoms. The dosage of OROS-MPH was titrated monthly, reaching 72 mg/day as a maintenance dose, and ADHD symptoms improved after 4 months of treatment. The patient was followed up regularly for 7 years during his OROS-MPH treatment. No adverse effects were reported, including stimulant addiction. He was stable overall and functioned well in his daily activities. His pain never recurred.

**Conclusion:**

This case report suggests that MPH may be potentially effective in treating chronic pain. Further studies are needed to confirm whether MPH improved chronic pain simultaneously with or separately from the improvement in ADHD. Moreover, elucidating the anatomical sites and molecular pharmacological mechanisms related to the action of MPH in pain modulation and perception is essential. Such sites include the descending dopaminergic pain pathway and higher cortical areas. Furthering our understanding may reinforce the justification for treating chronic pain using MPH.

## 1. Introduction

Chronic pain is defined as pain that persists or recurs for more than 3 months and affects one or more anatomical regions. It is associated with significant psychological distress or functional disability in daily activity and cannot be explained by another underlying etiology (1). The prevalence is estimated to be 27.5% worldwide (2). In addition, almost half of all patients receive inadequate pain management, and more than half of treated patients respond poorly to pain medications (3).

Several studies have shown that difficult-to-treat chronic pain is often associated with mental disorders (4). Around 25.93% of survey respondents had experienced chronic pain while having a mental disorder during their lifetime (5). Attention deficit hyperactivity disorder (ADHD) is a common mental disorder that co-occurs with pain disorders (3, 5, 6), is characterized by inattentive and/or hyperactive and impulsive behaviors, and typically emerges in childhood and can persist until adulthood (7, 8). Adults with ADHD symptoms have a higher chance of experiencing non-specific pain (9), which is likely due to altered pain perception (10, 11), motor inhibition problems, and heightened muscle tone (12). Although the pathophysiology of both chronic pain and ADHD involves the neurochemistry of regions that mediate cognitive and emotional functions (13), the specific mechanisms remain unclear.

Many patients with ADHD worldwide are not being treated as early as they should. A population-based study reported that less than 50% of patients with ADHD were treated with medication (14). In comorbidity of ADHD and chronic pain, many patients may first present to the clinic with only pain complaints. Treatment that merely focuses on eliminating pain may obscure the diagnosis of ADHD. In addition, untreated ADHD can lead to worsened symptoms, including chronic pain, whereas optimal outcomes can only be achieved by treating all comorbidities that exist as contributors or consequences of chronic pain (15). The first-line treatment for adult patients with ADHD is psychostimulants with MPH as moderately potent in reducing ADHD symptoms (16–18). In addition to this property, some studies suggested stimulants, including MPH, may have antinociceptive effects (10, 19, 20). However, the MPH utility for pain has not been widely investigated.

This case report presents an adult patient with comorbid chronic pain and ADHD. Regular treatment with MPH for his newly established ADHD diagnosis led to an unexpected and dramatic improvement in his 15 years of chronic pain complaints within 1 month. To our knowledge, there has only been one similar report of a pediatric case (21). However, this is the first well-documented adult case of this association which follows the CARE Guidelines for reporting medical cases in the literature (22). We also discuss whether the effectiveness of MPH in treating pain regardless of ADHD comorbidity was likely coincidental or a potentially consistent phenomenon.

## 2. Case presentation

A 43-year-old, unemployed, unmarried, university graduated, Japanese male patient presented with chronic pain that had recurred and persisted for 15 years at our child and adolescent psychiatric outpatient department after being referred from an adult psychiatric unit for further ADHD examination in our tertiary hospital.

History of chronic pain symptoms included somatic complaints of chest pain, back pain, and headache, which occurred alternately, and sometimes epigastric pain and nausea. The pain was evaluated with a numeric rating scale (NRS) score, which ranged from 1 (no pain) to 10 (worst pain). Daily, his pain had ranged from 2/10 to 6/10, but mostly 5/10 on average. The patient had histories of anxiety and depression that preceded pain and received antidepressants at a primary hospital. At the onset of pain, the patient visited a primary healthcare clinic due to chest pain, but the medical examinations revealed no abnormalities.

Consequently, he visited various clinics and received various examinations and medications for the pain. However, none of the treatments provided optimal outcomes. He was referred to a psychiatrist in a primary hospital and was also prescribed immediate-release MPH (IR-MPH) because of suspected ADHD comorbidity. With IR-MPH, the patient reported dramatic improvement in his pain symptoms with an NRS score of 0/10 and discontinued the visit. When the pain symptoms recurred, he visited the same hospital and received IR-MPH again, reducing the pain to an NRS of 3/10 on average. However, IR-MPH was discontinued following Japan’s drug distribution regulation change, and his pain worsened. Subsequently, he was referred to an anesthesiologist and received oral gabapentin and epidural block therapy, neither of which improved his pain.

He was referred to an adult psychiatrist in our tertiary hospital. Physical examination, electroencephalography (EEG), and head magnetic resonance imaging (MRI) showed no abnormalities. The patient insisted on having MPH treatment because he believed it would improve his pain based on his experience. However, his attending psychiatrist suspected an MPH dependency and thus rejected his request. The following year, he was hospitalized because his global assessment of functioning (GAF) score was 38–40, with chronic pain and depression. Following discharge, the physician recommended cognitive behavioral therapy (CBT), but he refused. Subsequently, he consented to modified electroconvulsive therapy (m-ECT), but his pain worsened after the eighth m-ECT session. Therefore, it was discontinued.

Throughout his medication history, he ever received acetaminophen, non-opioid analgesics, and various combination of antidepressants. However, combinations of two or three drugs did not adequately alleviate his pain, and MPH is an exception. The report on detailed medication history could not be obtained from previous physicians, clinics, and hospitals.

There was no family history of chronic pain or neurodevelopmental disorders. The psychosocial history indicated that during childhood, the patient was forgetful, careless, and had difficulty organizing schoolwork tasks. He sometimes forgot his school bag when he went to school. He was not good at maintaining social behavior and had been called a ‘strange child’. However, his parents overlooked his behavioral problems. His condition continued into adulthood, during which he often changed jobs, was reprimanded for his inadvertent mistakes, and had relational conflicts in the workplace. Regarding the psychological aspects of this case, especially coping strategies, the patient prefers treatment with a clear scientific basis, and willingly received treatments such as drug therapy, nerve blocks, and ECT for his chronic pain. On the other hand, the patient thought that psychosocial treatments were ineffective, such as he refused cognitive-behavioral therapy.

**Figure 1:**
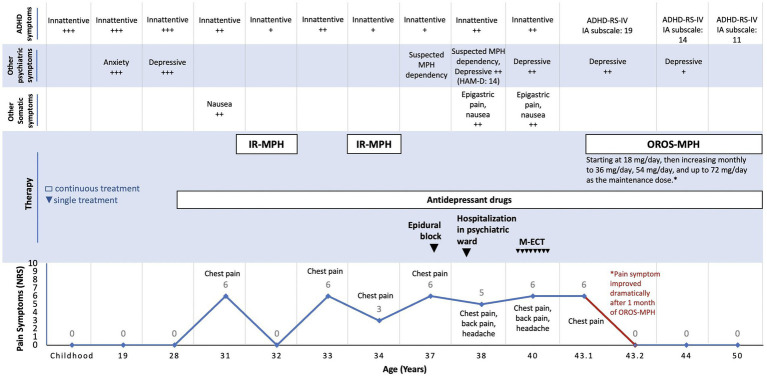
Timeline of clinical symptoms and therapies. ADHD, attention deficit hyperactivity disorder; NRS, numeric rating scales for pain; MPH, methylphenidate; IR-MPH, immediate-release methylphenidate; OROS-MPH, osmotic-release oral system methylphenidate; HAM-D, Hamilton depression rating scale; M-ECT, modified electroconvulsive therapy; ADHD-RS-IV, ADHD rating scale IV; IA, inattentive.

When we examined the patient, his NRS was 5/10, with predominantly chest pain. Current physical, electrocardiography, chest radiography, and laboratory examinations revealed no abnormalities. Thus, we suspected that his type of chronic pain was idiopathic. A thorough psychiatric examination led by a child and adolescent psychiatrist resulted in a diagnosis of ADHD. The patient met the Diagnostic and Statistical Manual of Mental Disorders (DSM)-IV criteria for ADHD, predominantly inattentive type, in which eight symptoms of inattention had persisted for 15 years. We used the ADHD Rating Scale (RS)-IV (23) with slight adjustments of items to suit an adult patient. The inattention subscale score was 19. The delay in the firm diagnosis of ADHD in this patient was likely due to the solely inattentive type, overlooked impact in life, and masked by pain comorbidity. The Wechsler Adult Intelligence Scale-III (24) scores consisted of verbal intelligence quotient (VIQ), performance IQ (PIQ), and full scale IQ (FIQ) were 130, 86, and 106, respectively. Figure 1 shows a timeline of his clinical symptoms and treatments.

Following the ADHD diagnosis, OROS-MPH was started at a titrated dose of 18 mg/day, increasing monthly to 36 mg/day, 54 mg/day, and up to 72 mg/day as the optimal maintenance dose. In addition, we treated his recurrent depressive episodes with amoxapine 100 mg/day. Flunitrazepam 2 mg/day and etizolam 0.25 mg/day were also prescribed for each of his secondary insomnia and anxiety symptoms in combination or separately when necessary.

After 1 month of OROS-MPH treatment, he reported no more pain with an NRS score of 0/10 and could resume light activities. After 4 months, he reported chronic pain alleviation and inattentive symptoms improvement at work. His ADHD-RS-IV inattention subscale score was 14.

The patient has been followed up for the last 7 years and is currently in ‘peak performance’ condition, functioning well in daily activities and attending employment support facilities twice per week. His current GAF score is 55, his ADHD-RS-IV inattention subscale score is 11, and his NRS score is 0/10. Regular addiction evaluation using the DSM-5 criteria showed no evidence of stimulant use disorder, and the patient adhered to the recommended use and dosage of MPH and indicated no self-medications. The regular evaluation through anamnesis indicated that the patient could tolerate MPH well, no adverse effects were reported, and his pain never recurred.

## 3. Discussion

Adult patients with ADHD often initially present to the clinic with comorbid symptoms, which results in difficulties in clearly differentiating ADHD during early assessments (25). Moreover, not only delayed diagnosis but also treatment is often seen in clinical practice (14). In this case, the prolonged treatments focused on eliminating pain may have obscured ADHD symptoms and caused delayed ADHD treatment. Increasing evidence suggests that ADHD patients experience chronic pain throughout life because they tend to be more sensitive to it (9–11, 26). Moreover, a previous study reported that ADHD increases the risk of chronic pain (9), and another study reported that 72.5% of adult patients with chronic pain are newly diagnosed with ADHD (27). Previous reports have discussed the association between MPH and pain (10, 11, 21, 28). Some studies only reported MPH effects for altering the pain perception of ADHD patients without chronic pain (10, 11), focused on pediatric and adolescent patients (11, 21), and addressed the risks of stimulant and opioid abuse in ADHD patients related to pain (28). However, although from a single case, this report differs from existing literature where we have newly reported the potent effect of MPH in eliminating chronic pain in an adult patient with ADHD. In this case, chronic pain may have been a comorbid condition of ADHD or a completely independent condition. Regardless, MPH was effective in treating this condition. We offer several potential mechanisms in this regard.

Regarding neurotransmission in chronic pain, persistent alterations may occur in the descending modulatory pain pathway without physical abnormalities (29). This pathway is mediated by monoamine neurotransmitters, such as serotonin (5-HT), norepinephrine (NE), and dopamine (DA), and inhibits or facilitates pain stimuli transmission at the dorsal horn level (30). Following the application of an acute pain stimulus, the descending pain circuitry adjusts its plasticity according to its ability to increase pain inhibition. However, disruption to this inhibitory mechanism results in persistent pain (29). In contrast to 5-HT and NE, the role of DA in the descending monoaminergic pain pathway is poorly understood.

Evidence has shown that the descending dopaminergic pathway projecting from the hypothalamic A11 nucleus to the spinal dorsal horn or spinal trigeminal nucleus caudalis plays a role in pain modulation (31), where activation of the A11 nucleus is mediated by D2-like receptors that induce an anti-nociceptive effect (32). A study has reported that decreased DA levels likely contribute to chronic pain symptoms in Parkinson’s disease (33), and levodopa may also be effective in alleviating polyneuropathic pain (34). Some studies using positron emission tomography found that patients with chronic pain syndromes may be characterized by deficits in presynaptic DA activity (35) and tonic DA levels (36) or disruptions in DA responses to noxious stimulation (37). Given these findings, this patient may have had a DA imbalance that simultaneously caused ADHD and chronic pain due to descending dopaminergic pain pathway disruptions (38, 39).

Oral MPH significantly increases extracellular DA in the brain by blockade of the DA transporter, which inhibits reuptake and triggers the amplification of released DA (40). Although we did not find evidence of direct MPH action sites along the descending pain pathways, we speculate that MPH increases DA, activating the A11 nucleus to inhibit noxious stimuli. This effect is mediated by D2 receptors, which have a higher affinity to low DA concentrations (30). In addition, A11 lies adjacent to A10 in the ventral tegmental area (VTA) of the hypothalamus and is a neuroanatomical site of MPH action (41). DA may increase because MPH inhibits DA reuptake by blocking NE transporters, which have a high affinity for DA in specific brain regions (42). There was also a case of an adult patient with ADHD and generalized pain from fibromyalgia syndrome who responded dramatically to atomoxetine, a selective NE reuptake inhibitor, with a 60% pain reduction (43). However, as NE and 5-HT increase descending pain inhibition (30), antidepressant drugs possessing properties that inhibit 5-HT and NE reuptake initially prescribed to this patient did not alleviate the pain. Therefore, this patient’s pain was likely related primarily to the descending dopaminergic pathway.

From a functional neuroimaging perspective, the descending pain pathway integrates cognitive and emotional outputs from higher cortical areas, such as the anterior cingulate cortex (ACC), insular cortex (IC), and amygdala (29). It is suggested that the mesolimbic DA pathway, which is involved in the reward system, is essential in pain modulation and perception (44–46), which may be impaired in ADHD (38). Furthermore, the amygdala is an essential structure in pain integration (47), and the IC contributes to the affective-motivational aspects of pain sensory-discriminative functions and simultaneously activates cognitive areas, such as the prefrontal cortex (PFC) and ACC, to shape pain perception (48). These areas are hyperactivated in ADHD patients in response to negative emotional stimuli (49), including pain. Moreover, alterations in gray matter density, connectivity, and activity in mesolimbic areas, PFC, and ACC are characteristics of both chronic pain (50, 51) and ADHD (52). In ADHD patients, MPH improves the hypodopaminergic state of the PFC and limbic system (53) and increases DA activity in the parietal cortex, PFC, and striatum, which regulate cognitive functions (54, 55). Therefore, MPH may indirectly improve the integration of cognitive and emotional outputs during pain modulation and perception, particularly in the abovementioned brain regions.

Although MPH use as an analgesic for pain treatment remains uncommon and off-label, several studies have investigated its antinociceptive effect. A double-blind, randomized controlled study showed that the antinociceptive effect of MPH significantly increases the cold pain threshold and tolerance in healthy men (19). In addition, a study in traumatic brain injury patients reported a slight decrease in pain intensity following a standard dose of MPH (20). Another study reported that in one of two patients undergoing ongoing opioid treatment for cancer, the pain intensity and frequency decreased during a 3-week observation with MPH combination therapy (56). Other studies have reported that ADHD is less prevalent than other mental disorders, such as depression, anxiety, and substance use disorders, in chronic pain populations (5, 57, 58). In this patient, chronic pain improved dramatically within 1 month, followed by improvement in attention after 4 months. The varied course of improvement suggests that the mechanisms underlying the effect of MPH in treating chronic pain and ADHD may be independent.

Although potentially effective, the use of MPH requires caution because of the side effects of abuse and addiction (59). The benefits of offering patients MPH as a primary therapy must outweigh the risks. Although this patient was initially suspected of having an MPH addiction, the course of illness and substantial long-term pain management ruled out addiction. Apart from that, viewing this case from the psychological aspect, another possibility to consider is the psychological nature of placebo analgesia. However, the placebo effect could also be physiological, in which the dopaminergic systems are likely implicated in placebo pain relief (60). In addition, because ADHD is a comorbidity in this case, thus a dopaminergic system abnormality is still more likely to be suspected. However, assuming the placebo effect, we cannot further provide the scientific explanation that MPH exclusively exhibited such an effective outcome compared to other treatments previously given.

We acknowledge the limitation that this report is merely based on a single case whose outcome may not be generalized to all cases. Although we have shown and discussed the possible mechanisms by which MPH alleviates chronic pain, current evidence is insufficient because of the comorbidity with ADHD. Thus further research in this field is required. In addition, we could not obtain precise and detailed information entirely about the diagnosis and medication before the patient was referred to us. However, the information presented by the patient was sufficient to understand the course of the disease.

In conclusion, MPH may be potentially effective in treating chronic pain. It is suggested that physicians should be aware of a potential ADHD diagnosis when managing patients with chronic pain with inadequate response to treatment. Conversely, in the case of MPH independently alleviating chronic pain, randomized control trial (RCT) is needed in patients with pain without ADHD, following the result of a previous RCT study that reported the antinociceptive effect of MPH, which significantly increased the threshold and tolerance to cold pain in healthy subjects (19). Finally, determining the anatomical sites and molecular pharmacological mechanisms underlying the effect of MPH on pain modulation and perception, such as the descending dopaminergic pain pathway and higher cortical areas, may justify treating chronic pain using MPH.

## 4. Patient perspective

Before treatment, the patient described pain mainly in his chest. At its worst, his pain lasted all day, and he was sometimes unable to sleep. While in pain, he was unable to concentrate on daily activities. After OROS-MPH treatment, the pain disappeared completely. His pain symptoms never recurred, even on days when he forgot to take OROS-MPH.

Furthermore, his ADHD symptoms improved. He makes fewer mistakes, is less forgetful, and can concentrate on tasks better. He does not experience the short-term craving-like sensations with OROS-MPH that he experienced with IR-MPH and feels better about taking it long-term without side effects. Overall, he considers OROS-MPH a necessary drug.

## Data availability statement

The original contributions presented in the study are included in the article/supplementary material, further inquiries can be directed to the corresponding author.

## Ethics statement

Ethical approval was not provided for this study on human participants because this case report does not require ethical or institutional review board approval, and only written informed consent is required from the patient for the publication of this paper. We obtained written informed consent from the patient for the publication of this case report with confidentiality by removing any identifiable data.

## Author contributions

EZ and AS conceptualized the study, collected medical record data, and wrote the manuscript. EZ performed the literature research review. AS was the lead treating clinician. JE and TS reviewed and supervised the manuscript. All authors contributed to and approved the final version.

## Funding

This study was supported by a research fund (156195-J15F0001) commissioned by the Niigata Prefectural Hospital Bureau to the Department of Community Psychiatric Medicine, Niigata University Graduate School of Medical and Dental Sciences.

## Conflict of interest

The authors declare that the research was conducted in the absence of any commercial or financial relationships that could be construed as a potential conflict of interest.

The reviewer VP-S declared a past co-authorship with the author EZ to the handling editor.

## Publisher’s note

All claims expressed in this article are solely those of the authors and do not necessarily represent those of their affiliated organizations, or those of the publisher, the editors and the reviewers. Any product that may be evaluated in this article, or claim that may be made by its manufacturer, is not guaranteed or endorsed by the publisher.
